# The Story of the Insane from Year to Year

**Published:** 1897-09-11

**Authors:** 


### THE STORY OF THE INSANE FROM YEAR TO YEAR.

Tenth Series.

### THE EAST RIDING ASYLUM AT BEVERLEY.

The numbers were almost stationary here, being 363 on January 1st and 367 on December 31st. There were 57 first admissions, 7 readmissions, 25 recoveries, and 30 deaths. The recoveries stand at 40·98 per cent. of the admissions and the deaths at 8·13 per cent. of the average number resident, the former being a little above the average and the latter below it. Eleven of the deaths were caused by cerebral and spinal diseases, 4 by consumption, and 1 by pneumonia. Intemperance in drink is the assigned cause of the insanity in 5 of the year's admissions, and hereditary tendency in 28. Considerable additions are now being made to the female wing of the asylum. The average weekly cost per head is 7s. 7½d., a low rate for so small an asylum. There are 11 private patients, who pay from 13s. to 30s. a week.

### BELFAST DISTRICT ASYLUM.

The limits of accommodation are given at 400; but there were 810 patients in residence on January 1st, 1896, and by the end of the year the number had risen to 859. There were 212 first admissions, 61 readmissions, 134 recoveries, 43 discharged relieved, and 44 deaths. The recoveries stand at 49 per cent. of the admissions, and the deaths at 5·3 per cent. on the average number resident. Of the deaths, 28 were caused by cerebral and spinal diseases (only 1 of these being from general paralysis) and 3 from consumption. To various moral causes are ascribed 94 of the admissions, intemperance in drink figures in 27, and hereditary influence in 28. There is unquestionably considerable over-crowding, and yet the above figures show that the recovery rate is very high and the death rate very low. An old mansion at Purdysburn has been converted into a temporary asylum for 120 cases, and it is proposed to build an entirely new asylum on the estate. The annual cost per head was £23 18s. 8d.

